# Detection of *Ochyromera ligustri* (Coleoptera: Curculionidae: Curculioninae) in *Ligustrum* spp. (Oleaceae) Using Newly Developed PCR Primers

**DOI:** 10.3390/insects15050320

**Published:** 2024-04-30

**Authors:** Ilgoo Kang, Amy Roda, Brandi Misiaszek, Tanner Sparks, Rodrigo Diaz

**Affiliations:** 1 Department of Entomology, Louisiana State University Agricultural Center, Baton Rouge, LA 70803, USA; ikang1@lsu.edu (I.K.); bmisiaszek@agcenter.lsu.edu (B.M.); tsparks@agcenter.lsu.edu (T.S.); 2 Department of Entomology, College of Ecology and Environmental Science, Kyungpook National University, Sangju 37228, Gyeongsangbuk-do, Republic of Korea; 3 Animal and Plant Health Inspection Service, Plant Protection and Quarantine, United States Department of Agriculture, Miami, FL 33158, USA; amy.l.roda@usda.gov

**Keywords:** classical biological control, invasive pest, molecular detection, polymerase chain reaction, privets

## Abstract

**Simple Summary:**

Privets (*Ligustrum* spp.) are popular hedge shrubs that escape from gardens and cause negative impacts by invading forests in the United States. The narrow host specificity of the seed-feeding weevil, *Ochyromera ligustri*, suggests that this weevil could be considered a fortuitous biological control agent of privets. To find out the host range of this weevil, time-consuming experiments involving rearing and dissections are often conducted. Herein, we developed PCR primers to rapidly and accurately detect the presence of the weevil in privet fruits. We confirmed that the novel PCR primers specifically work for *O*. *ligustri* even with a small quantity of DNA, which was 0.01 ng. Using the newly developed PCR-based detection saves time and labor in research related to the detection and impact of this weevil.

**Abstract:**

*Ligustrum* spp. (Oleaceae) have become invasive species in the US and negatively affect native plant diversity and richness in forests. *Ochyromera ligustri* (Coleoptera: Curculionidae) is considered a potential biological control agent in the US because adults feed on the foliage and larvae are seed-feeders of *Ligustrum* spp. To discover the relationships between *O*. *ligustri* and *Ligustrum* spp., fruit dissections or rearing and field observations are required. In the current research project, novel PCR primers were developed to rapidly detect the DNA of *O*. *ligustri* in molecular analyses without rearing and observation. The developed PCR primers worked even with 0.01 ng of DNA and did not amplify the DNA of the other five curculionid species tested. When the novel primers were tested with three *Ligustrum* spp. species common in the southeastern US, the DNA of *O*. *ligustri* was detected from all three species. We expect that the novel primers will be utilized to find out the presence and impact of *O*. *ligustri* on *Ligustrum* spp rapidly and accurately.

## 1. Introduction

Ligustrinae Koehne 1893 is a monophyletic subtribe comprising *Ligustrum* (Oleaceae; common name: Privet) and *Syringa* (Oleaceae; common name: Lilac) [1]. Many members of the two genera are ornamental shrubs, and *Ligustrum* includes species frequently used as hedge plants [2]. Since the late 18th century, at least nine species of *Ligustrum* have been introduced into the United States (US) for ornamental purposes [3], and various hybrids and varieties have also been developed [4]. Planted *Ligustrum* spp. escape from gardens to the wild, and they are considered invasive species or potential problem species in the US [3].

The invasion of *Ligustrum sinense* in the US has resulted in negative impacts on forest ecosystems [5]. The imported plants were especially well-established in the southeastern US [3], and many native plants and shrub species have been replaced by *L*. *sinense* in floodplains [6]. Moreover, the aggressiveness to *L. sinense* thickets has been associated with a reduction in forest regeneration, suggesting a change in ecosystem communities [7]. In the forests near the Wolf River in Tennessee, where the river was channelized, resulting in dry areas, *L. sinense* became more abundant in the post-channelized conditions. Additionally, a noticeable deceleration in the growth of canopy oaks was observed [8]. The growth of other native species commonly distributed in floodplain forests, *Acer negundo* L. subspe. *negundo* (Sapindaceae; common name: box elder), *Boehmeria cylindrica* (L.) Swartz (Urticaceae; common name: false nettle), *Carex tribuloides* Wahlenburg var. tribuloides (Cyperaceae; common name: blunt broom sedge), and *Chasmanthium latifolium* (Michaux) Yates (Poaceae: common name: northern wood oats), were also inhibited by *L. sinense* in floodplain forests along the Piedmont ecoregion of South Carolina [6].

To manage the populations of *L*. *sinense* and confirm the usefulness of conventional control methods for the plants, numerous studies have been conducted. The effectiveness of physical and chemical controls was compared, and chemical treatment was more successful than physical treatment [9]. Among chemicals, glyphosate was more effective than triclopyr, but the former did not provide 100% control of *L*. *sinense* populations from the test plots [10]. In a study investigating the effectiveness of burning and chemical treatments, neither treatment method was effective in reducing *L*. *sinense* populations [11]. Due to the negative environmental effects and other limitations of conventional control methods, biological control has been considered as an alternative method for the management of *L*. *sinense* and some other *Ligustrum* species [12,13,14].

**Figure 1:**
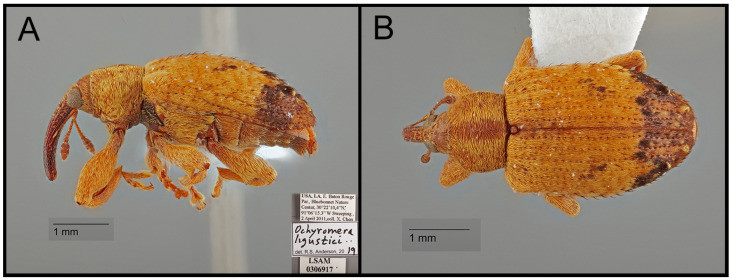
*Ochyromera ligustri*. (**A**) lateral habitus; (**B**) dorsal habitus.

In June 1959, the seed-feeding weevil *Ochyromera ligustri* Warner 1961 (Figure 1) was first detected in Wake County, North Carolina, US, and was confirmed to be feeding on *L. japonicum* Thunberg 1780 [15,16]. The adults of this weevil are 3.0 to 4.7 mm in body length and feed on the fruits of *L*. *amurense* Carrière, 1861 [15,16], *L*. *japonicum* [15,16], *L*. *lucidum* WT Aiton, 1810 (Warner 1961) [15,16], *L*. *sinense* [15,16,17], *Syringa* spp., and *Vitis* spp. (Vitaceae; common name: grapes) [16]. Even though the exact origin of *O. ligustri* is unknown, the oriental or far east Paleartic regions are considered the weevil’s origin [15,17]. After its first discovery in North Carolina, *O*. *ligustri* has been reported from most states in the southeastern US, New Jersey, and North and South Dakota (Figure S1) [15,16,18,19]. In North Carolina, adult females were noted to oviposit a single egg in the fruit seeds or flesh, and the emerging larvae feed on the fruit and seeds during the fall and winter [16]. The larvae mature by April, and adult weevils emerge from the fruit found on the ground below the privets in May [16]. The complete development of *O. ligustri* was confirmed from only *L*. *japonicum* [16,17], but *O*. *ligustri* in the US was presumed to be adapted to *L. sinense* and be able to complete its development [20]. These previous investigations were conducted based on observation, rearing, and dissection, which are time-consuming steps to determine host associations [21]. Due to the uncertainty of the host associations from these reports, it is important that future studies define the host plants suitable for adult feeding and immature development [22].

With the advancement of molecular techniques, diverse molecular analyses are being utilized in biological control research. In particular, polymerase chain reaction (PCR)-based detection methods are widely used to identify target organisms [23,24,25], confirm host ranges and trophic relationships [26,27,28,29], and detect gut content [30,31]. Since the traditional rearing and dissection of insects are laborious and lengthy experimental processes, the utilization of PCR-based methods allow investigators to rapidly detect and correctly identify target organisms [32,33,34]. In a tachinid parasitoid study, a PCR-based method identified parasitism rates that were three times higher than rearing methods [34].

We propose to utilize a PCR-based method since molecular-based work can improve on the weakness of traditional rearing and dissection experimental methods, which are time-consuming, laborious, and error-prone. The potential of *O. ligustri* as a fortuitous biological control agent (BCA) of invasive *Ligustrum* spp. or a pest of the Oleaceae species is unknown. Therefore, the objectives of this study were (a) to develop species-specific PCR primers for the quick detection and identification of *O*. *ligustri* and (b) to confirm the host range of *O*. *ligustri* based on the molecular data.

## 2. Materials and Methods

### 2.1. Sample Collection and Processing

Multiple adults of *Ochyromera ligustri* were collected from *Ligustrum sinense* found along hiking trails at the Forest Community Park located in the southeastern part of East Baton Rouge Parish (Baton Rouge, LO, USA; site A) on 6 August 2022, using a beating sheet by the corresponding author. Fruit samples of *Ligustrum sinense* were collected at the same place on 4 October 2022. Weevil specimens were directly preserved in 100% ethanol. At the collecting site, *L. sinense* was the most abundant among *Ligustrum* spp., and *L. lucidum* was the second most abundant.

**Figure 2:**
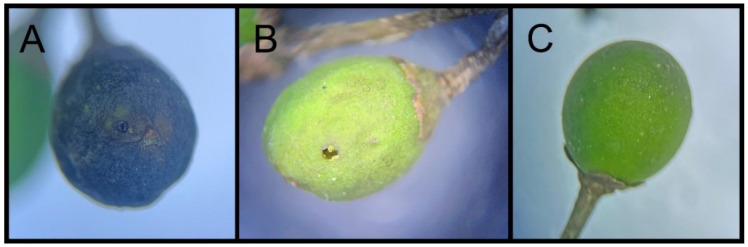
*Ligustrum sinensis* fruits of different conditions: (**A**) group i (unhealthy brown); (**B**) group ii (green with feeding or oviposition marks); (**C**) group iii (green without any feeding or oviposition marks).

We targeted seemingly unhealthy fruit samples or samples with feeding or oviposition marks by *O*. *ligustri*. The fruit samples were separated into three groups using a stereomicroscope: i. unhealthy brown; ii. green with feeding or oviposition marks; iii. green without any feeding or oviposition marks (Figure 2). Fruit samples of *L. lucidum* and *L. japonicum* were collected near the University Lake at Louisiana State University (Baton Rouge, Louisiana; site B) and forest on a private property (Rosedale, Louisiana; site C). Fruit samples of non-*Ligustrum* spp. having a similar fruit shape living near *L*. *sinense* on site A were collected by the authors. Fruits of the following plant species were included: *Ardisia crenata* Sims, 1818 (Primulaceae; common name: coral ardisia), *Ilex vomitoria* Aiton (Aquifoliaceae; common name: yaupon holly), *Nandina domestica* Thunberg (Berberidaceae; common name: heavenly bamboo), and *Sambucus canadensis* L. (Adoxaceae; common name: American black elderberry). We preserved the fruit samples in a refrigerator at 4 °C. The fruit samples were used in molecular analyses within two weeks of collection.

For a primer specificity test, specimens of five other curculionid species were included. Specimens of *Anthonomus quadrigibbus* Say, 1831, *Tychius picirostris* (Fabricius, 1787), and *Lignyodes* sp. were provided by the Louisiana State Arthropod Museum (LSAM; Baton Rouge, LO, USA), and specimens of *Cyrtobagous salviniae* Calder and Sands, 1985 and *Tanysphyrus lemnae* (Paykull, 1792) were reared in an experimental pond in St. Gabriel, Louisiana by the LSU Entomology Laboratory and included in the test. Five to ten live adults of *C. salviniae* and *T. lemnae* were transferred into vials containing 70% ethanol and preserved until processing. To confirm the effectivity of the primers on *O. ligustri* collected outside of Louisiana, weevils were collected in 2023 from Wesson, Mississippi, and Auburn, Alabama; and *L. sinense* berries were collected from Kathleen, Perry, and Thomaston, Georgia; and Houston, Beaumont, and High Island, Texas. Weevil specimens were preserved in 100% ethanol, while the berry samples were stored in a freezer at −20 °C until used for processing.

### 2.2. Primer Design

To develop the COI species-specific primers, a LepF1/LepR1 primer set was used to obtain a 658 bp-long COI sequence of *Ochyromera ligustri* (Table 1). After obtaining the COI sequence of *O. ligustri*, we searched for similar sequences using the MegaBLAST algorithm [35] using the nucleotide basic local alignment search tool (BLAST) (http://www.ncbi.nlm.nih.gov/BLAST/; accessed on 19 October 2022) [36] and compared the COI sequence of *O. ligustri* with ten other most similar weevil COI sequences (GenBank access numbers: MK397247.1; JN298259.1; JF889041.1; HQ560690.1.; KX774487.1; KP421266.1; KX087271.1; MN171279.1; MK758421.1; and ON553604.1) using the NCBI multiple sequence alignment viewer (MSA) (https://www.ncbi.nlm.nih.gov/projects/msaviewer/; accessed on 19 October 2022), and suitable regions for species-specific forward and reverse primers were found (Figures S2 and S3). The relative positions of the newly developed primers are illustrated (Figure S6). The Eurofins oligo analysis tool (https://eurofinsgenomics.com/en/resources/tools/oligo-analysis/; accessed on 19 October 2022) [37] was utilized to confirm the properties of the selected primer sequences and the possibility of use in a polymerase chain reaction (PCR). After confirming the results from the online tool, the primers were ordered.

### 2.3. DNA Extraction and Polymerase Chain Reaction Amplification

The DNeasy Blood and Tissue kit (Qiagen, Hilden, Germany) was used to extract the genomic DNA from the weevils and the weevil DNA in Chinese privet fruits. The entire weevil body (one to three individuals) was ground using Fisherbrand™ RNase-Free Disposable Pellet Pestles (Fisher Scientific, Carlsbad, CA, USA) in 180 µL of ATL buffer and 20 µL of proteinase K solution (Qiagen, Hilden, Germany). For the fruit samples, we put one to ten fruit samples in each microcentrifuge tube (1.5 mL). When we used one to two fruit samples, we ground the fruit samples using pestles, and 180 µL of ATL buffer and 20 µL of proteinase K solution or 1.5 times of each buffer were used (Table S1). When we put ten fruit samples in a microcentrifuge tube, we did not grind the fruit samples but cut each fruit sample in half instead. A total of 900 µL of ATL buffer and 100 µL of proteinase K solution was used for a tube including ten fruit samples (Table S1). We incubated the prepared samples overnight at 56 °C. The tubes with weevils or one to two fruit samples were directly used in the remaining steps to extract the DNA following the manufacturer’s protocol. For the tube with ten fruit samples, ~400 µL of the incubated supernatant was relocated to new microcentrifuge tubes and processed (Table S1; tubes 6, 8, and 10). In the following two steps, 100 µL of AL buffer (Qiagen, Hilden, Germany) and 100 µL of 100% ethanol were used (Table S1). In the remaining steps, we followed the manufacturer’s protocol.

The PCR reaction was performed using a T100™ thermal cycler (Bio-Rad, Hercules, CA, USA). The total volume of each PCR sample was 25 µL, containing 12.5 µL of DreamTaq Green PCR Master Mix (2X) (Thermo Scientific, Waltham, MA, USA), 1–2 µL of template genomic DNA, 7.5–8.5 µL of nuclease-free water, 0.5 µL MgCl2 (Thermo Scientific), and 1.0 µL of each primer at 5 µM resuspended in a low TE buffer (10 mM Tris, 0.05 mM EDTA, pH 8). Using a gradient PCR method ranging from 45 to 65 °C, an optimal annealing temperature for a newly developed primer set was found. A PTC-200 thermal cycler was used (MJ Research, Watertown, MA, USA). The PCR conditions were 95 °C for 3 min; 35 cycles of 95 °C for 30 s, 58 °C for 30 s, and 72 °C for 1 min; and a final extension at 72 °C for 7 min for a newly developed species-specific primer set. For LepF1 and LepR1, the PCR conditions were 95 °C for 3 min; 5 cycles of 95 °C for 30 s, 45 °C for 30 s, and 72 °C for 1 min; and 35 cycles of 95 °C for 30 s, 51 °C for 30 s, and 72 °C for 1 min; and a final extension at 72 °C for 7 min. Using a 2.0% agarose gel stained with 1X SYBR™ Safe DNA Gel Stain (Invitrogen, Carlsbad, CA, USA), we confirmed the amplified products. The PCR products were cleaned using a primer depletion clean-up method developed at the LSU genomics facility (Baton Rouge, LA, USA) and sequenced on the 3130xl genetic analyzers (Applied Biosystems, Foster City, CA, USA) using BigDye Terminator v 3.1 chemistry (Applied Biosystems) at the same facility.

### 2.4. DNA Sequence Assembly

Using Geneious Prime (version 2022.0.1) (https://www.geneious.com; accessed on 2 September 2022) [39], we obtained the DNA sequences with high quality, which were edited. The primer regions were confirmed and trimmed. The edited sequences were assembled via de novo assembly. The consensus sequences were compared with the sequences deposited in the GenBank database using BLASTn.

### 2.5. Primer Specificity

The DNA from the other five curculionid species was extracted using the method described in 2.3. To confirm the success of DNA extraction, ~650 bp of the COI region was amplified using LepF1/LepR1 and visualized on a 2.0% stained agarose gel. We then tested the newly developed primers in PCR and gel electrophoresis using the DNA extracted from *O. ligustri* and the other five curculionid species to see whether the primer set only amplified the DNA of *O. ligustri*. In addition, the PCR products amplified using the newly developed primers were sequenced and assembled using the same method mentioned above to confirm whether the DNA was amplified from *O. ligustri*.

### 2.6. Primer Sensitivity

To confirm the sensitivity of the newly developed primer set, we ran a PCR amplification with different quantities of DNA extracted from *O*. *ligustri*. After confirming a raw DNA sample concentration (5.05 ng/µL) using the Qubit™ 4 fluorometer using the Qubit™ dsDNA BR Assay Kit (Thermo Fisher Scientific, Waltham, MA, USA), we then prepared eight DNA samples for the sensitivity test (10 ng, 5 ng, 1 ng, 0.5 ng, 0.1 ng, 0.05 ng, 0.01 ng, and negative). For the samples with 10 ng and 5 ng, we loaded 2 µL and 1 µL into 25 μL PCR samples, respectively. For the samples with ≤ 1 ng DNA concentrations, we diluted the raw DNA sample (5.05 ng/µL) with nuclease-free water and prepared the appropriate DNA concentrations. In the negative control, only nuclease-free water was loaded. After 35 cycles of the PCR reaction, the PCR samples were visualized on a 2.0% stained gel.

### 2.7. Application to Fruit Samples of Ligustrum spp.

To confirm the ability of the newly developed primers to detect *O. ligustri* DNA in the infected fruits of *L. sinense* in the PCR and gel electrophoresis, we prepared the fruit samples following the method in 2.1 and extracted the DNA using the method described in 2.3 (Table S1). We amplified the DNA of *O*. *ligustri* with the species-specific primers in the PCR and confirmed the presence of the DNA of *O*. *ligustri* as well as the ability of the species-specific primers in gel electrophoresis. After we confirmed that the novel primers amplified the DNA of O. ligustri in the fruit samples of L. sinense, we utilized the primers to determine if the DNA of *O*. *ligusti* was present in the fruits of *L*. *lucidum* and *L*. *japonicum* (Table S2). To test for *O. ligustri*’s DNA presence in the fruit samples of the other plant species having similar fruit shapes and sizes living near *L*. *sinense* in site A, the same molecular methods were used (Table S3).

### 2.8. Distribution Map

Distribution records of *Ligustrum* spp. (31,392), *Syringa* spp. (14,615), and *O. ligustri* (123) in the US were available from the Global Biodiversity Information Facility (GBIF) (26 June 2023). We downloaded all GPS coordinate data from the GBIF and generated a map presenting the distribution of *Ligustrum* spp., *Syringa* spp., and *O. ligustri* in the US using ArcGIS Pro 3.0 (version 3.0.0) [40] (Figure S1).

## 3. Results

### 3.1. Species Primer Design and Optimal PCR Conditions

A species-specific primer set, COI_O_ligustri_F1/COI_O_ligustri_R1 (Table 1), was successful in detecting *O. ligustri* DNA in the tested fruits. The primer set produced 182 bp of a target sequence after trimming the primer regions (Figure S4). The obtained 182 bp sequence (sequences with the following GenBank accession numbers: OQ199864; OQ199865) was a 100% match with an internal region of the 658 bp sequence of *O*. *ligustri* (sequences with the following GenBank accession numbers: OQ199862; OQ199863). Using a gradient PCR, we found that the optimal annealing temperature for the species-specific primers was 58 °C (Figure S4) when the T-100 thermal cycler (Bio-Rad) was used.

### 3.2. Primer Specificity

**Figure 3:**
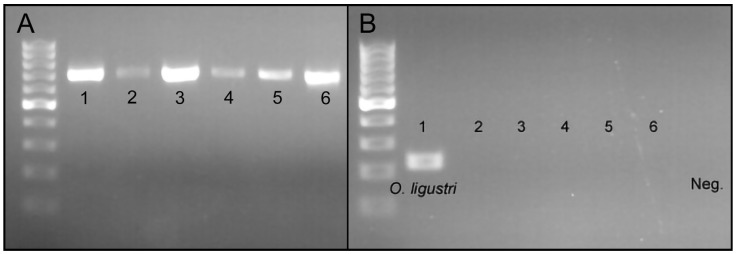
(**A**) Amplified COI sequences (658bp) of 1. *O. Ligustri*, 2. *A. Quadrigibbus*, 3. *Ta. Lemnae*, 4. *Lignyodes* sp., 5. *Ty. picirostris*, 6. *C. salviniae.* (**B**) The primer specificity test results indicate that the newly developed species-specific primers only worked for *O. ligustri*. 1. *O. ligustri*, 2. *A. quadrigibbus*, 3. *Ta. lemnae*, 4. *Lignyodes* sp., 5. *Ty. picirostris*, and 6. *C. salviniae*.

In order to confirm whether the raw DNA of *O. ligustri*, *A. quadrigibbus*, *Ta. lemnae*, *Lignyodes* sp., *Ty. Picirostris*, and *C. salviniae* were successfully extracted from the specimens, we amplified the standard COI region (658-bp) of the six curculionid species and ran a gel (Figure 3A). The COI DNA sequences of all curculionid species were successfully amplified and obtained (Figure 3). Using the raw DNA of the six curculionid species, we tested the specificity of the newly developed PCR primers (COI_O_ligustri_F1 and COI_O_ligustri_R1). The novel PCR primers only amplified 182 bp of *O. ligustri* DNA (Figure 3B).

### 3.3. Primer Sensitivity

**Figure 4:**
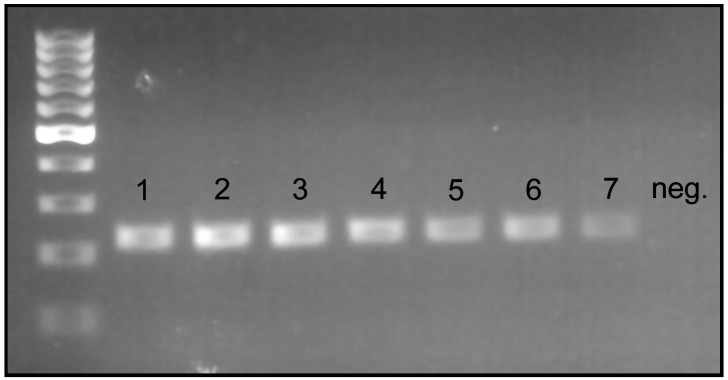
Primer sensitivity test results (35 cycles); 1. 10 ng; 2. 5 ng; 3. 1 ng; 4. 0.5 ng; 5. 0.1 ng; 6. 0.05 ng; 7. 0.01 ng.

In the primer sensitivity test, we confirmed that strong visible bands were produced when ≥0.05 ng of raw DNA was used in a 25 µL PCR reaction (35 cycles), and a slightly visible band was produced with 0.01 ng of raw DNA in the PCR reaction with the same conditions (Figure 4).

### 3.4. Application to the Fruit Samples of Ligustrum spp.

**Figure 5:**
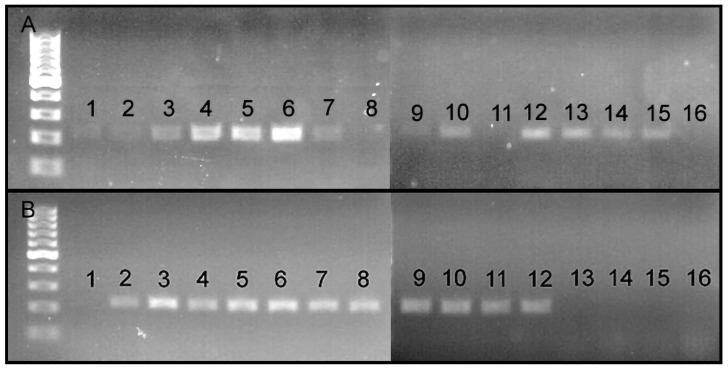
(**A**) The host range test results of *O. ligustri* indicated that the DNA of *O. ligustri* was detected from the three *Ligustrum* spp. species: detected in *L*. *sinense* at sites A and C (1, 2, 3, 4, 5, and 6); detected in *L. lucidum* at sites B and C (7, 9, 10 and 12); detected in *L. japonicum* at site B (13, 14, and 15); absent in *L. lucidum* at sites B and C (8 and 11). Detailed experimental setup from Table S2. (**B**) DNA detection results of *O*. *ligustri* from the fruit samples of *L*. *sinense*: detected at site A in the samples (2, 3, 4, 5, 6, 7, 8, 9, 10, 11, and 12); not detected at site A in some samples (1, 13, 14, and 15), detailed experimental set up included in Table S1.

**Figure 6:**
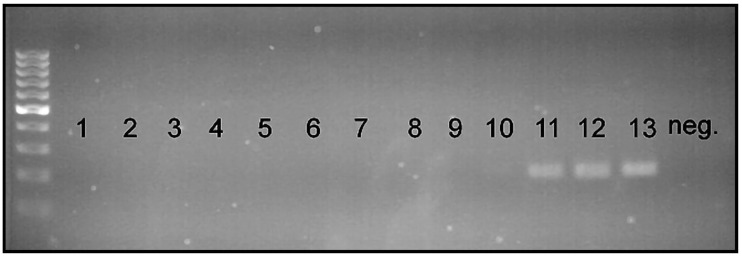
DNA detection results of *O. ligustri* from *Ardisia crenata*, *Sambucus* sp., Unidentified sp., *Nandina* sp., and *L. sinense*: only detected from *L. sinense* in 11, 12, and 13. The detailed experimental setup is included in Table S3.

The DNA of *O. ligustri* was detected in the fruit samples of *L. sinense*, especially from the fruit group i (brown and unhealthy) and group ii (green with feeding or oviposition marks), but not from the fruit group iii (healthy and green without any marks) (Figure 5B). The DNA of *O*. *ligustri* was found in all three *Ligustrum* species included in the test (*L. sinense*, *L. lucidum*, and *L. japonicum*) (Figure 5A). The DNA of *O*. *ligustri* was only detected from *L. sinense*, but not from the other non-ligustrum species collected in site A, including *Ardisia crenata*, *Sambucus* sp., unidentified sp., and *Nandina* sp., which have fruits with a similar shape and size (Figure 6). The primers were also successful in detecting the DNA in weevils from both MS and AL and in berries from MS and GA (Figure S5).

## 4. Discussion

The newly developed primers successfully amplified the DNA of *O. ligustri*, not only from the weevils but also from the *O. ligustri* larvae-infested fruit samples of all three *Ligustrum* spp. species (*L. sinense*, *L. lucidum*, and *L. japonicum*) (Figure 5 and Figure 6). All the steps (sample preparation, DNA extraction, PCR, gel electrophoresis) were completed in two days. The novel primer set for *O*. *ligustri* will be useful for the rapid and accurate identification and detection of *O*. *ligustri*, enabling future studies to rapidly confirm the presence of *O*. *ligustri* in the newly infested regions and measure the initial dispersal and establishment rates of *O. ligustri*.

Researchers can save time in confirming the presence of *O*. *ligustri* with direct PCR or time-efficient DNA extraction methods. However, sensitivity tests should be performed when raw DNA are extracted using different isolation methods. To detect the specific DNA from a targeted organism, finding the optimal PCR conditions is important for specificity and efficiency [41]. Our gradient PCR results showed that the novel primer set visualized fairly bright bands at an annealing temperature range of 46–64 °C (Figure S4). However, we recommend the use of annealing temperatures of 55–58 °C because (a) the brightest bands were produced at 55 °C and 58 °C, (b) low annealing temperatures frequently produce non-specific bands [42], (c) band production is decreased when high annealing temperatures are used [42]. Regarding the PCR cycles, we confirmed that 40 PCR cycles produced a brighter band compared to 35 PCR cycles when 0.01 ng of DNA of *O*. *ligustri* was used. However, using more than 40 PCR cycles is undesirable due to the possibility of producing non-specific bands [43].

The newly developed PCR primers can be employed for the streamlined confirmation of the DNA presence of *O*. *ligustri* in *Ligustum* spp. and *Syringa* spp. established in the US. The widespread distribution of *Ligustum* spp. and *Syringia* sp. in the central southeastern and northeastern US, respectively (Figure S1), emphasizes the importance of understanding the impact of *O. ligustri* across this vast climatic gradient. We envision utilizing the novel primers to conduct a citizen science project to determine the current distribution of *O. ligustri* in the US. The specific primers will allow the prompt testing of fruit samples for the presence of weevil DNA within one week.

While these PCR primers facilitate swift and straightforward *O. ligustri* DNA detection from fruit samples, gauging the actual infection rates necessitates suitable sampling strategies. Drawing from the approach of diverse studies [44,45,46,47,48], the refinement of the optimal sampling and measurement strategies is crucial to assess the impact of *O*. *ligustri* on *Ligustrum* spp. For expeditious and cost-effective *O*. *ligustri* screening, the adoption of pooled testing, akin to SARS-CoV-2 (COVID-19) monitoring [47,49,50], holds promise. Implementing pooled testing entails gathering seemingly unhealthy fruit, enhancing the detection of *O*. *ligustri* DNA, and ultimately economizing both the time and expenses associated with screening processes.

We also acknowledge certain limitations of the developed method. Specifically, identifying the sex of *O*. *ligustri* and discerning whether the extracted weevil DNA originated from adults or juveniles (such as eggs and larvae) presented challenges. Furthermore, confirming whether this weevil uses *Ligustrum* spp. as their oviposition fruit proved challenging. Even though we did succeed in detecting weevil DNA on or within the fruit of the three *Ligustrum* spp. species, confirming the actual *O. ligustri* oviposition remained unclear since the DNA detected in the fruits could have originated from saliva, feces, or body parts. Conventional rearing and observational experiments are needed in order to ascertain the sex of *O*. *ligustri* and confirm the emergence of adults from the fruit. X-ray scanning, as suggested by Karunakaran et al. [51], can be an alternative method to confirm which plant species are used as the oviposition sites of *O. ligustri*.

No *O. ligustri* DNA was detected from the non-*Ligustrum* spp. included in the current study. However, the detection of DNA in the fruits of various *Ligustrum* spp. suggests that *O. ligustri* juveniles might use these species. Therefore, we suggest conducting additional in vitro and field tests that include closely related species in Oleaceae as well as other species possessing fruit with similar sizes and shapes to determine the actual host range of *O*. *ligustri*. Since several *Ligustrum* spp. species are considered ornamental due to their foliage, further studies should emphasize not only immature development but also adult feeding on the foliage.

## Figures and Tables

**Table 1:** Primer information used in this project. LepF1/LepR1 was used to obtain the standard COI DNA sequences of the curculionid species, and COI_O_ligustri_F1/COI_O_ligustri_R1 were the newly developed species-specific primers for *O. ligustri* in the current project.

Gene	Primer Name and Direction	Sequence (5′ → 3′)	Source
COI	LepF1 (F)	ATTCAACCAATCATAAAGATATTGG	[38]
COI	LepR1 (R)	TAAACTTCTGGATGTCCAAAAAATCA	[38]
COI	COI_O_ligustri_F1 (F)	TTACTACCTCCTTCACTAATTTTACTTC	Newly developed
COI	COI_O_ligustri_R1 (R)	CCGCTCTAGTGTCATTCCTAT	Newly developed

## Data Availability

All the analyzed data are publicly available at the NCBI nucleotide database: www.ncbi.nlm.nih.gov/genbank/ (accessed on 23 January 2023).

## Supplementary Materials

The following supporting information can be downloaded at: https://www.mdpi.com/article/10.3390/insects15050320/s1, Figure S1. Distribution map of *O. ligustri*, *Ligustrum* spp., and *Syringa* spp. in the US. Green squares represent *O. ligustri*, gray dots represent *Ligustrum* spp., and black triangles represent *Syringa* spp.; Figure S2. Gene region of a newly developed forward COI primer within 658-bp sequence of *O. ligustri* (263 to 289). MSA viewer showed the gene region is interspecifically highly diverse (red color); Figure S3. Gene region of a newly developed reverse COI primer within 658-bp of of *O. ligustri* (473 to 494). MSA viewer showed the gene region is interspecifically highly diverse (red color); Figure S4: Gradient PCR results indicated that the newly developed species-specific primers worked best at the 58 °C annealing temperature; Figure S5: DNA detection results of *O. ligustri* from fruits and adults weevils: 1: *L. sinense* fruits (n = 10; Houston Arboretum, Texas; absent); 2: *L. sinense* fruits (n = 10, High Isle, Texas; absent) 3: *L. sinense* fruits (n = 5; Tyrell, Texas; absent); 4: *L. sinense* fruits (n = 5; Wesson, Mississippi; detected); 5: *L. sinense* berries (n = 10; Kathleen, Georgia; detected); 6: *L. sinense* fruits (n = 10; Sprewell Bluff, Georgia; absent); 7: *L. sinense* fruits (n = 5; Perry, Georgia; detected); 8: *O. ligustri* adult (n = 1; Wesson, Mississippi; detected); 9: *O. ligustri* adult (n = 1; Wesson, Mississippi; detected); 10: *O. ligustri* adult (n = 1; Auburn, Alabama; detected); 11: *O. ligustri* adult (n = 1; Auburn, Alabama; detected); 12: *O. ligustri* adult (n = 1; Louisiana, control 1; detected); 13: *O. ligustri* adult (n = 1; Louisiana, control 2; detected); 14: *O. ligustri* adult (n = 1; Louisiana, control 3; detected); Figure S6: Positions of the newly developed primers on COI DNA barcoding region; Table S1: Experimental setup to determine presence of *O*. *ligustri* DNA in *L. sinense* fruits in Louisiana; Table S2: Experimental setup to determine presence of *O*. *ligustri* DNA in three *Ligustrum* spp., *L. sinense* (Ch), *L. lucidum* (Tr) and *L. japonicum* (Ja) fruits in Louisiana; Table S3: Experimental setup to determine presence of *O*. *ligustri* DNA in fruits of non-ligustrum plants, *Ardisia crenata* (Ca), *Sambucus* sp. (El), *Ilex vomitoria* (IL), *Nandina* sp. (Na), *L. sinense* (Ch).
