# Supplementary material for: Detection of Ochyromera ligustri (Coleoptera: Curculionidae: Curculioninae) in Ligustrum spp. (Oleaceae) Using Newly Developed PCR Primers
Source: Insects. 2024 Apr 30;15(5):320. doi: 10.3390/insects15050320 (PMC11122171; doi:10.3390/insects15050320)
Supplement: Supplementary file 1 [file insects-15-00320-s001.zip › insects-2926602-supplementary.pdf]

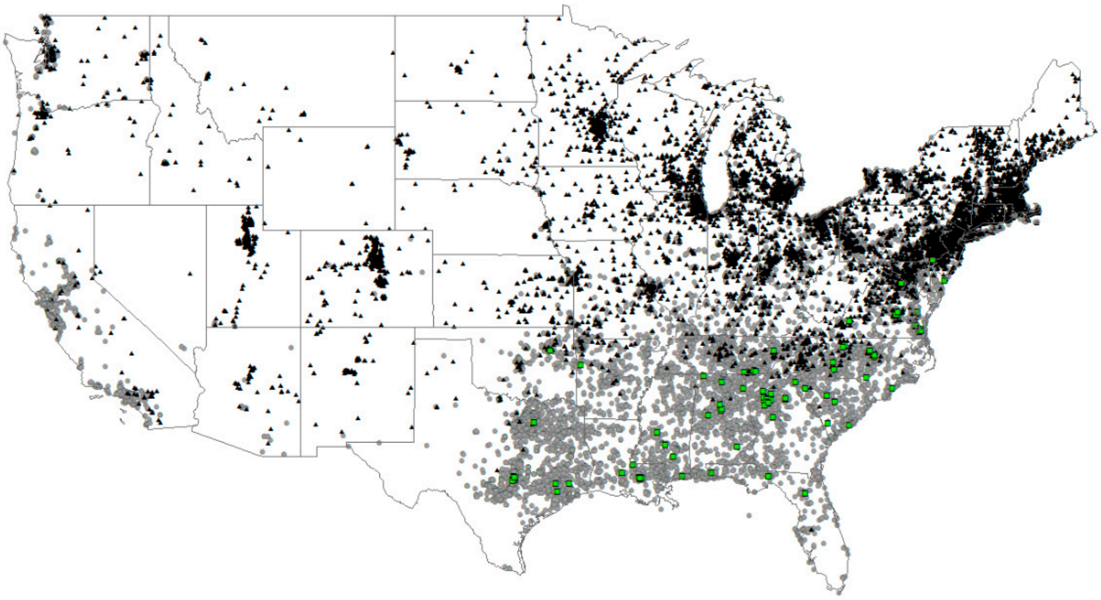

Figure S1. Distribution map of *O. ligustri*, *Ligustrum* spp., and *Syringa* spp. in the US. Green squares represent *O. ligustri*, gray dots represent *Ligustrum* spp., and black triangles represent *Syringa* spp.

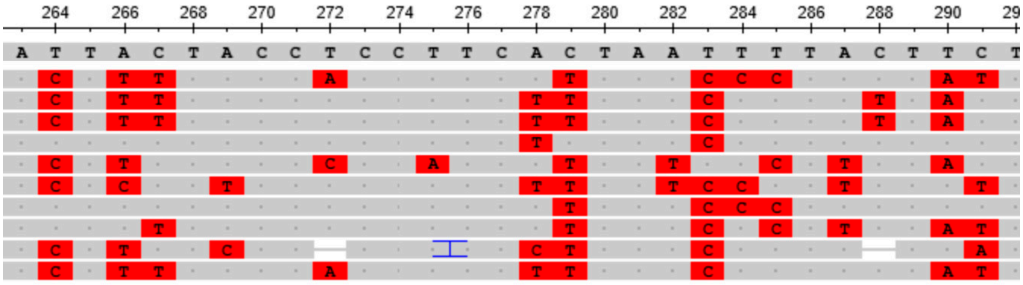

Figure S2. Gene region of a newly developed forward COI primer within 658-bp sequence of *O. ligustri* (263 to 289). MSA viewer showed the gene region is interspecifically highly diverse (red color).

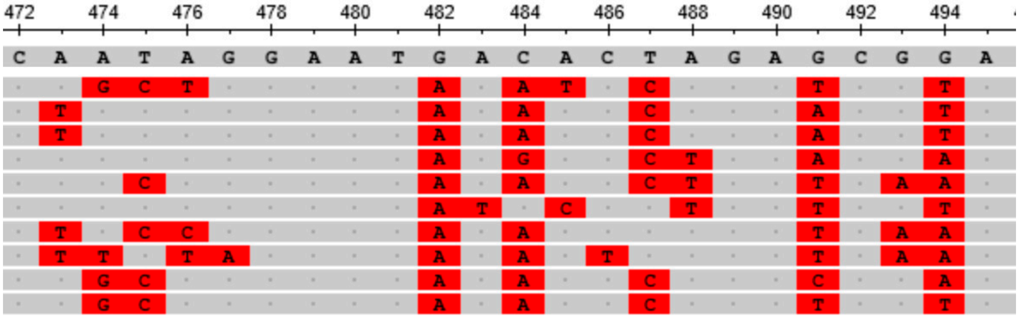

Figure S3. Gene region of a newly developed reverse COI primer within 658-bp of of *O. ligustri* (473 to 494). MSA viewer showed the gene region is interspecifically highly diverse (red color).

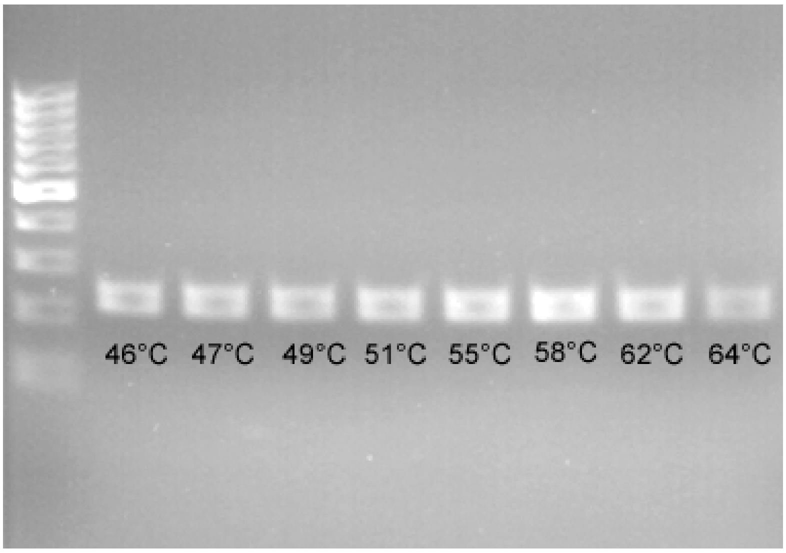

Figure S4. Gradient PCR results indicated that the newly developed species-specific primers worked best at the 58°C annealing temperature.

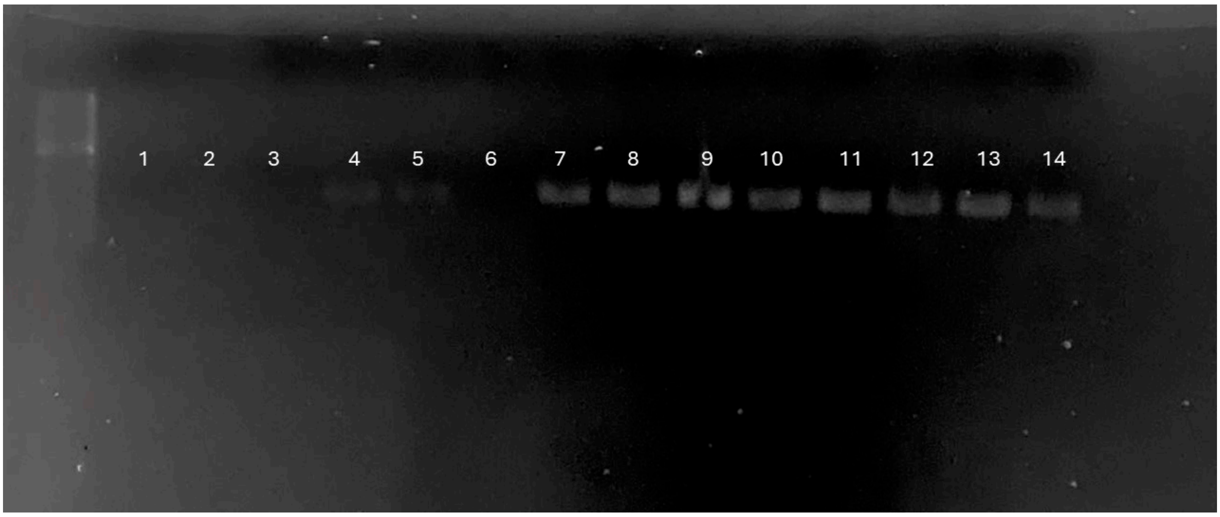

Figure S5. DNA detection results of *O. ligustri* from fruits and adults weevils: 1: *L. sinense* fruits (n =10; Houston Arboretum, Texas; absent); 2: *L. sinense* fruits (n = 10, High Isle, Texas; absent) 3: *L. sinense* fruits (n = 5; Tyrell, Texas; absent); 4: *L. sinense* fruits (n =5; Wesson, Mississippi; detected); 5: *L. sinense* berries (n = 10; Kathleen, Georgia; detected); 6: *L. sinense* fruits (n = 10; Sprewell Bluff, Georgia; absent); 7: *L. sinense* fruits (n = 5; Perry, Georgia; detected); 8: *O. ligustri* adult (n = 1; Wesson, Mississippi; detected); 9: *O. ligustri* adult (n = 1; Wesson, Mississippi; detected); 10: *O. ligustri* adult (n = 1; Auburn, Alabama; detected); 11: : *O. ligustri* adult (n = 1; Auburn, Alabama; detected); 12: *O. ligustri* adult (n = 1; Louisiana, control 1; detected); 13: : *O. ligustri* adult (n = 1; Louisiana, control 2; detected); 14: *O. ligustri* adult (n = 1; Louisiana, control 3; detected).

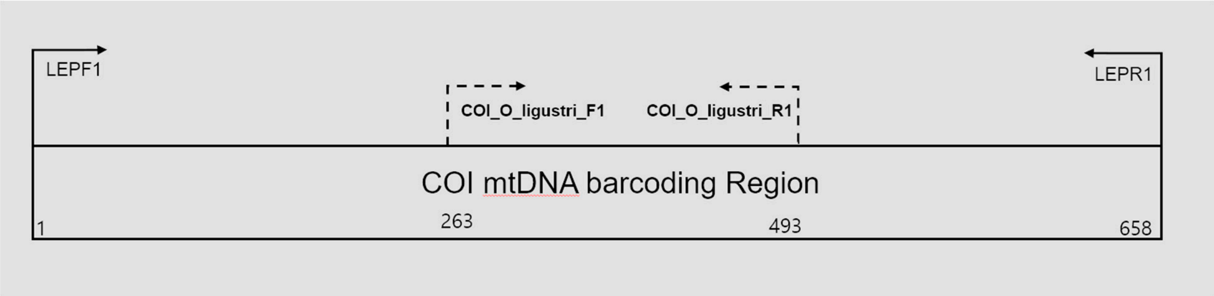

Figure S6. Positions of the newly developed primers on COI DNA barcoding region.

Table S1. Experimental setup to determine presence of *O. ligustri* DNA in *L. sinense* fruits in Louisiana.

	# 1	# 2	# 3	# 4	# 5	# 6	# 7	# 8	# 9	# 10	# 11	# 12	# 13	# 14	# 15	# 16
**Number of fruits**	1	2	1	1	10	10	10	10	10	10	10	10	10	10	10	10
**ATL buffer (μL)**	180	180	270	270	900	900	900	900	900	900	900	900	900	900	900	900
**Proteinase K solution (μL)**	20	20	30	30	100	100	100	100	100	100	100	100	100	100	100	100
**AL Buffer (μL)**	200	200	200	200	100	100	100	100	100	100	100	100	100	100	100	100
**100% EtOH (μL)**	200	200	200	200	100	100	100	100	100	100	100	100	100	100	100	100
**Fruit group**	i	i	i	i	ii	ii	ii	ii	i	i	i	i	iii	iii	iii	iii
**Plant species**	Ch	Ch	Ch	Ch	Ch	Ch	Ch	Ch	Ch	Ch	Ch	Ch	Ch	Ch	Ch	Ch
**Site**	A	A	A	A	A	A	A	A	A	A	A	A	A	A	A	A

Table S2. Experimental setup to determine presence of *O. ligustri* DNA in three *Ligustrum* spp., *L. sinense* (Ch), *L. lucidum* (Tr) and *L. japonicum* (Ja) fruits in Louisiana.

	# 1	# 2	# 3	# 4	# 5	# 6	# 7	# 8	# 9	# 10	# 11	# 12	# 13	# 14	# 15
**Number of fruits**	1	2	10	1	2	10	1	2	4	1	2	5	1	2	10
**ATL buffer (μL)**	180	180	900	180	180	900	180	180	900	180	180	900	180	180	900
**Proteinase K solution (μL)**	20	20	100	20	20	100	20	20	100	20	20	100	20	20	100
**AL Buffer (μL)**	200	200	100	200	200	100	200	200	100	200	200	100	200	200	100
**100% EtOH (μL)**	200	200	100	200	200	100	200	200	100	200	200	100	200	200	100
**Fruit group**	i & ii	i & ii	i & ii	i & ii	i & ii	i & ii	i & ii	i & ii	i & ii	i & ii	i & ii	i & ii	i & ii	i & ii	i & ii
**Plant species**	Ch	Ch	Ch	Ch	Ch	Ch	Tr	Tr	Tr	Tr	Tr	Tr	Ja	Ja	Ja
**Site**	A	A	A	C	C	C	C	C	C	B	B	B	B	B	B

Table S3. Experimental setup to determine presence of *O. ligustri* DNA in fruits of non-ligustrum plants, *Ardisia crenata* (Ca), *Sambucus* sp. (El), *Ilex vomitoria* (IL), *Nandina* sp. (Na), *L. sinense* (Ch).

	# 1	# 2	# 3	# 4	# 5	# 6	# 7	# 8	# 9	# 10	# 11	# 12	# 13
**Number of fruits**	10	10	10	10	10	10	4	4	10	1	2	10	10
**ATL buffer (μL)**	900	900	900	900	900	900	900	900	900	180	180	900	900

**Proteinase K solution (μL)**	100	100	100	100	100	100	100	100	100	20	20	100	100
**AL Buffer (μL)**	100	100	100	100	100	100	100	100	100	200	200	100	100
**100% EtOH (μL)**	100	100	100	100	100	100	100	100	100	200	200	100	100
**Fruit group**	iii	iii	i & ii	i & ii	Dark purple	Dark purple	ii & iii	ii & iii	ii & iii	i	i	i	ii
**Plant species**	Ca	Ca	Ca	Ca	El	El	IL	IL	Na	Ch	Ch	Ch	Ch
**Site**	A	A	A	A	A	A	A	A	A	A	A	A	A
